# Three-Dimensional Radiomics Features From Multi-Parameter MRI Combined With Clinical Characteristics Predict Postoperative Cerebral Edema Exacerbation in Patients With Meningioma

**DOI:** 10.3389/fonc.2021.625220

**Published:** 2021-04-15

**Authors:** Bing Xiao, Yanghua Fan, Zhe Zhang, Zilong Tan, Huan Yang, Wei Tu, Lei Wu, Xiaoli Shen, Hua Guo, Zhen Wu, Xingen Zhu

**Affiliations:** ^1^ Department of Neurosurgery, Second Affiliated Hospital of Nanchang University, Nanchang, China; ^2^ Department of Neurosurgery, Beijing Tiantan Hospital, Capital Medical University, Beijing, China

**Keywords:** radiomics, meningioma, cerebral edema exacerbation, machine learning, MRI

## Abstract

**Background:**

Postoperative cerebral edema is common in patients with meningioma. It is of great clinical significance to predict the postoperative cerebral edema exacerbation (CEE) for the development of individual treatment programs in patients with meningioma.

**Objective:**

To evaluate the value of three-dimensional radiomics Features from Multi-Parameter MRI in predicting the postoperative CEE in patients with meningioma.

**Methods:**

A total of 136 meningioma patients with complete clinical and radiological data were collected for this retrospective study, and they were randomly divided into primary and validation cohorts. Three-dimensional radiomics features were extracted from multisequence MR images, and then screened through Wilcoxon rank sum test, elastic net and recursive feature elimination algorithms. A radiomics signature was established based support vector machine method. By combining clinical with the radiomics signature, a clin-radiomics combined model was constructed for individual CEE prediction.

**Results:**

Three significance radiomics features were selected to construct a radiomics signature, with areas under the curves (AUCs) of 0.86 and 0.800 in the primary and validation cohorts, respectively. Two clinical characteristics (peritumoral edema and tumor size) and radiomics signature were determined to establish the clin-radiomics combined model, with an AUC of 0.91 in the primary cohort and 0.83 in the validation cohort. The clin-radiomics combined model showed good discrimination, calibration, and clinically useful for postoperative CEE prediction.

**Conclusions:**

By integrating clinical characteristics with radiomics signature, the clin-radiomics combined model could assist in postoperative CEE prediction before surgery, and provide a basis for surgical treatment decisions in patients with meningioma.

## Introduction

Meningioma is the most common intracranial tumor. Most meningiomas occur in the intracranial region, and more than 90% of meningiomas show benign growth (1). The incidence of meningiomas is 2:1 for females: males, the peak age is 45 years old, and it is rare for children, many asymptomatic meningiomas are incidental findings. At present, surgery is the first-line treatment, most of them have good prognosis (2), peritumoral edema is a common concomitant symptom of meningioma, up to 60-67.4% (3), easily complicated by cerebral edema postoperatively. Brain edema can be generally divided into cytotoxic brain edema and vasogenic brain edema. Meningioma edema is mainly angiogenic. For patients without peritumoral edema before operation, severe brain edema occurs after operation. Brain edema near the functional area is aggravated after operation, which seriously affects the prognosis of patients and prolongs the hospitalization time of patients. Peritumoral edema is a leading cause of morbidity and mortality in patients with brain tumors (4). Uncontrolled cerebral edema may result in refractory intracranial hypertension (RICH), and also leads to severe neurological deficits and potentially fatal herniation (5, 6). In a retrospective study, they evaluated the clinical and surgical records of 376 consecutive patients who underwent microsurgical removal of intracranial meningiomas between January 1995 and January 2001. 13 patients (3.5%) who met the following criteria were included for further analysis: CT scan or MR imaging showed increased extensive brain swelling with neurological deterioration after operation, which required further treatment intervention, such as artificial ventilation, endotracheal intubation or decompressive craniectomy for several days, however, not all of the edema worsened to the extent of the need to perform further treatment intervention, and most of them can get through the edema by strengthening dehydration (7). Therefore, it is very important to establish relevant models to predict the postoperative cerebral edema exacerbation (CEE) in patients with meningioma, also known as aggravation of postoperative edema (7, 8), closely observe the changes of patient’s condition, regularly review the head CT, strengthen the rational use of dehydration drugs, glucocorticoids, and even remove bone flap, so as to formulate the corresponding treatment plan.

Radiomics is a new machine learning method, which can extract data reflecting important biological tissue characteristics from medical image information (9). Compared with the traditional methods, the data mining of radiomics has two unique advantages (10). First of all, it allows semi-automatic or automatic extraction of imaging features and provides rich data related to qualitative analysis. Secondly, by identifying different sub regions and defining the spatial complexity of the disease, high-dimensional imaging information can reveal the heterogeneity within a region.

Recent studies have shown that radiomics has broad application prospects in early screening, accurate diagnosis, grading and staging, molecular marker prediction, treatment and prognosis of central nervous system diseases, and is helpful to formulate individualized treatment strategies (11–13). Therefore, in this retrospective study, we aimed to develop a radiomics model based on the minimal radiomic feature set of MR images to predict the aggravation of brain edema after meningioma surgery.

## Materials and Methods

### Patients

A total of 136 patients with meningioma from the Second Affiliated Hospital of Nanchang University were included in our study. The inclusion criteria were as follows: 1) meningioma patients who underwent initial tumor resection surgery from 2017 to 2019 at the Second Affiliated Hospital of Nanchang University; 2) available information of postoperative edema; 3) available preoperative brain MRI examination; 4) complete clinical data; and 5) meningioma confirmed by postoperative pathological analysis.

The Ethical Review Committee of the Second Affiliated Hospital of Nanchang University approved the study design and protocol. All included patients were randomized to the primary cohort (n=90) and validation cohort (n=46). The primary cohort was used for model construction, while the validation cohort was used for model internal validation.

### Clinical Characteristics

Eight preoperative clinical features from these patients were artificially collected: gender, age, peritumoral edema (negative or positive), tumor size (<2cm, 2-5cm or >5cm), tumor location (parasinoidal, facies convexa, skull base or others), hypertension (negative or positive), diabetes (negative or positive), and epilepsy (negative or positive).

One postoperative clinical feature CEE was artificially collected, CEE was defined as CEE can be defined if it meets any of the following criteria (8): 1) New sheet or finger brain edema occurs after operation, and the maximum diameter of edema is not less than 2cm; 2) If there is no peritumoral edema before operation, flaky, finger shaped or annular brain edema occurs after operation, and the maximum diameter of the tumor cavity in the same layer of the tumor or operation area is not less than 2 cm before operation or on the first day after operation. 3, if there is peritumoral edema before operation, the maximum diameter of lamellar, finger like or annular brain edema after operation is not less than 2 cm compared with the maximum diameter of brain edema on the same plane before operation or on the first day after operation.

### Brain MRI Sequence and Regions of Interest Delineating

**Figure 1:**
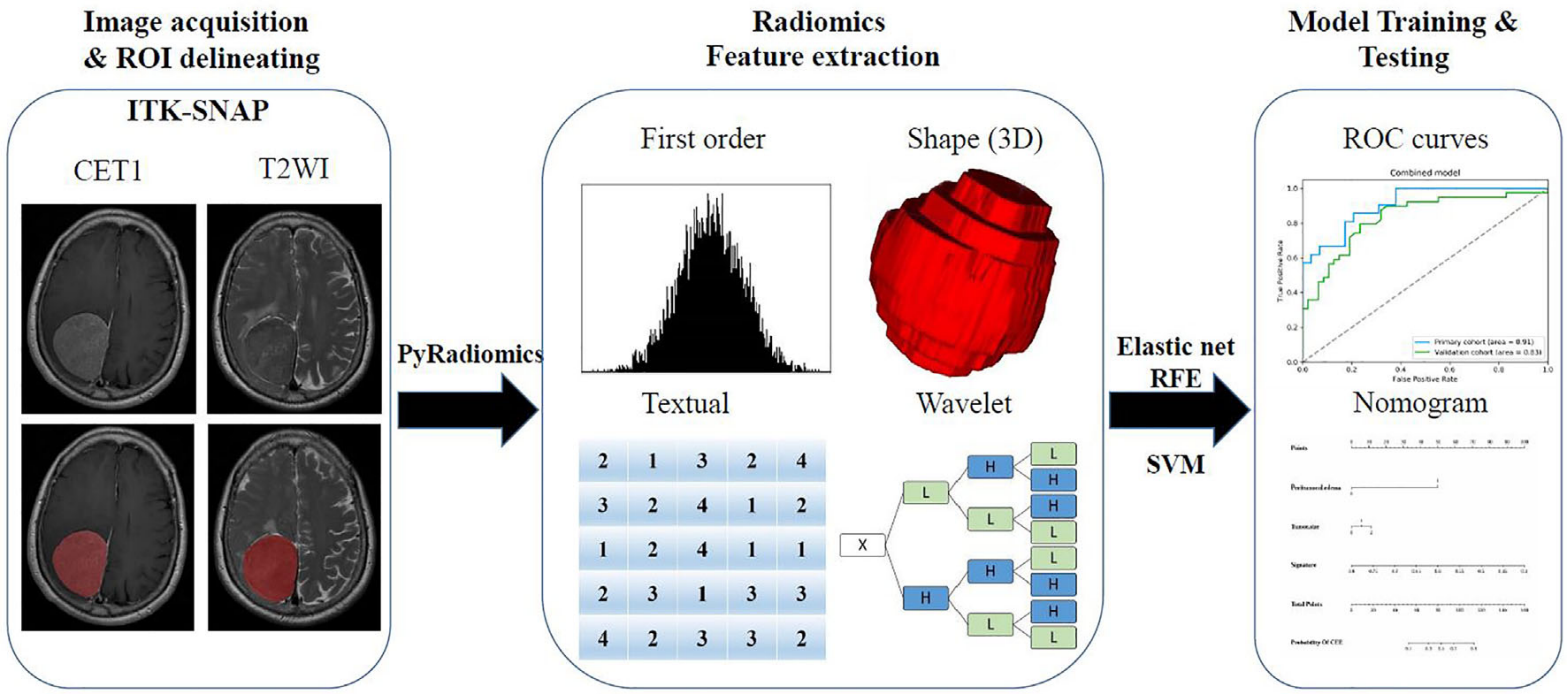
The flow chart of the present study. (I) Brain axial contrast-enhanced T1WI (CET1) T2-weighted imaging (T2WI) and MR images acquisition; Regions of interest (ROI) segmentation by ITK-SNAP software. (II) Four categories radiomics features extracted by PyRadiomics algorithm. (III) Radiomics Feature selection by elastic net and RFE algorithm, and model training and testing.

A flowchart of this study is shown in **Figure 1**. All patients underwent brain T2-weighted imaging (T2WI) and contrast-enhanced T1WI (CET1) MR imaging before surgery. The acquisition parameters of T2WI sequence were as follows: repetition time/echo time of 3640/98 ms, acquisition matrix of 320 × 224, slice thickness of 5 mm. Meanwhile, the acquisition parameters of T1WI sequence were as follows: repetition time/echo time of 2070/26 ms, flip angle of 90°, acquisition matrix of 320 × 256, slice thickness of 5 mm. CET1 was carried out the T1WI sequence parameters after rapid injection of a gadolinium-DTPA contrast agent. T2WI and contrast-enhanced T1WI in the in axial plane were utilized, and all DICOM format images were collected based on the picture archiving and communication system of the Second Affiliated Hospital of Nanchang University.

A neuroradiologist with 9 years of experience in meningioma diagnosis was responsible for mapping the three-dimensional regions of interest (ROIs) of tumors on the MRI images using ITK-SNAP software (University of Pennsylvania, www.itk snap.org). Then another neuroradiologist with 15 years of experience manually confirmed the findings. Any disagreement between the two neuroradiologists was resolved through a neuroradiologist with 31 years of experience.

### Radiomics Feature Extraction

Then, quantitative radiomics features were extracted from these ROIs using PyRadiomics (https://github.com/Radiomics/pyradiomics) (14). Each sequence can extract a total of 1,562 features, and these features were normalized to a value of 0 to 1 and classified into four categories (15): shape and size features (n = 14), first-order features (n = 180), textural features (n = 680), and wavelet features (n = 688).

The four types of features were described as follows (16, 17): first-order statistics describe the distribution of voxel intensities within the brain MRI image through commonly used and basic metrics; the three-dimensional size and shape features were independent from the gray level intensity distribution in the ROI, and were calculated on the non-derived image and mask; the textural features describing patterns or the spatial distribution of voxel intensities, which were calculated from respectively gray level co-occurrence (GLCM) and gray level run-length (GLRLM) texture matrices; Wavelet transform effectively decouples textural information by decomposing the original image, in a similar manner as Fourier analysis, in low –and high-frequencies.

### Radiomics Features Selection and Radiomics Signature Construction

Due to the large number and high complexity of the radiomics features, we needed to perform a selection process to reduce overfitting (18). The selection method was conducted as previously described (15) to prioritize the features. In short, univariate analysis by Wilcoxon rank sum test was used to identify the differential radiomics features between patients with postoperative CEE and non-CEE. In addition, elastic net algorithm (19) was used to select the most informative features. Elastic net is a method combining least absolute shrinkage and selection operator (LASSO) and ridge regression. LASSO (20) is a commonly used high-dimensional data analysis method that can improve the prediction accuracy and interpretation ability. Finally, a recursive feature elimination (RFE) algorithm through five-fold cross-validation was used to identity the finally radiomics features.

A radiomics signature was established based on the radiomics features selected from the primary cohort through the support vector machine (SVM) method. At the same time, differences in the signature distribution between soft and firm tumors were compared between the two cohorts using a violin plot. A receiver operating characteristic (ROC) (21) curve was drawn to display the predictive value of the radiomics signature.

### Construction and Validation of Clinical and Clin-Radiomics Combined Model

Multivariable logistic regression analysis was applied to construct a clinical model based on all included clinical features. Then, to establish a more comprehensive and accurate model for predicting the postoperative CEE, a clin-radiomics combined model was constructed by combining the most valuable clinical features with the radiomics signature. Akaike information criterion (AIC) (22) were used to screen the most valuable clinical features. And the usage and structure of the combined model was presented as a nomogram. ROC curve analyses and area under the ROC curve (AUC) were performed to evaluate the discriminative efficacy of the clinical and clin-radiomics combined model in both the primary and validation cohorts.

### Calibration Curve Analysis and Decision Curve Analysis

Calibration curves and the Hosmer–Lemeshow test were used to assess the similarity between the predicted and observed postoperative CEE (23). Decision curve analysis was performed to evaluate the clinical application of the clin-radiomics combined model by quantifying the net benefits at different threshold probabilities (24).

### Statistical Analysis

A two-sided *P*-value of <0.05 was deemed to be statistically significant. The statistical software R (version 3.4.1, R Foundation for Statistical Computing, Vienna, Austria) was used to perform the statistical analysis. The calibration plot was analyzed with the ‘hdnom’ packages. The decision curve analysis was conducted by the function ‘dca.R’.

## Results

### Clinical Characteristics

In total, 207 patients with meningioma underwent neurosurgery at the Second Affiliated Hospital of Nanchang University from 2017 to 2019. After screening, 71 patients were excluded due to unavailable preoperative MRI data, excessive preoperative information loss or lack of postoperative information, or both. Ultimately, 136 patients with meningioma were identified and included in the study. The mean age at diagnosis was 54.169 ± 11.765 years, with a male-to-female ratio of 2.9:1 (101/35). Of the 136 patients, 45(33.1%) had a peritumoral edema. Postoperative CEE was present in 60 (44.1%) patients and non-CEE was present in 76 (55.9%) patients. All included clinical characteristics are summarized in **Table 1**.

**Table 1:** Patients’ characteristics of primary and validation cohorts.

Characteristics	Whole cohort (n=136)	Primary cohort (n=90)	Validation cohort (n=46)	P-value
**Gender**				
Male	101 (74.3%)	70 (77.8%)	31 (67.4%)	0.19
Female	35 (25.7%)	20 (22.2%)	15 (32.6%)	
**Age (year)**	54.169 ± 11.765	53.578 ± 11.237	55.236 ± 12.786	0.414
**Peritumoral edema**				
Negative	91 (66.9%)	62 (68.9%)	29 (63.0%)	0.493
Positive	45 (33.1%)	28 (31.1%)	17 (37.0%)	
**Tumor size**				
<2cm	18 (13.2%)	11 (12.2%)	7 (15.2%)	0.444
2-5cm	95 (69.9%)	66 (73.3%)	29 (63.1%)	
>5cm	23 (16.9%)	13 (14.5%)	10 (21.7%)	
**Location**				
Parasinoidal	37 (27.2%)	23 (25.6%)	14 (30.4%)	0.741
Facies convexa	28 (20.6%)	19 (21.1%)	9 (19.6%)	
Skull base	24 (17.6%)	18 (20.0%)	6 (13.0%)	
Others	47 (34.6%)	30 (33.3%)	17 (37.0%)	
**Hypertension**				
Negative	113 (83.1%)	74 (82.2%)	39 (84.8%)	0.706
Positive	23 (16.9%)	16 (17.8%)	7 (15.2%)	
**Diabetes**				
Negative	132 (97.1%)	86 (95.6%)	46 (100%)	0.147
Positive	4 (2.9%)	4 (4.4%)	0 (0%)	
**Epilepsy**				
Negative	134 (98.5%)	89 (98.9%)	45 (97.8%)	0.626
Positive	2 (1.5%)	1 (1.1%)	1 (2.2%)	
**Cerebral edema exacerbation**				
Negative	76 (55.9%)	55 (61.1%)	21 (45.7%)	0.086
Positive	60 (44.1%)	35 (38.9%)	25 (54.3%)	

Categorical variables were presented as the number (percentage). Continuous variables consistent with a normal distribution were presented as mean ± standard deviation, otherwise the median and quartile are used. Chi-Square or Fisher Exact tests, as appropriate, were used to compare the differences in categorical variables, while the independent sample t-test was used to compare the differences in continuous variables.

All patients were randomly divided into a primary cohort (n=90) and a validation cohort (n=46). There was no significant interclass difference in terms of gender, age, peritumoral edema, tumor size, tumor location, hypertension, diabetes, epilepsy, and postoperative CEE between the primary cohort and the validation cohort (**Table 1**, P=0.086–0.741). The results justify the use of the two datasets for training and testing.

### Correlation Between Postoperative CEE and Clinical Characteristics

As shown in **Table 2**, peritumoral edema, tumor size, and location showed significant relationships with postoperative CEE (P = 0.000–0.001). The results demonstrated that patients who had larger tumor size, peritumoral edema, parasinoidal and skull base tumor were more likely to have postoperative CEE. Conversely, we found no significant differences in gender, age, hypertension, diabetes, and epilepsy between the postoperative CEE and non-CEE groups (P = 0.076–0.810).

**Table 2:** Correlation between cerebral edema exacerbation and clinical characteristics of patients with meningioma in all patients.

Characteristics	All Patients (n=136)	Non-CEE (n=76)	CEE (n=60)	P-value
**Gender**				
Male	101 (74.3%)	59 (77.6%)	42 (70.0%)	0.312
Female	35 (25.7%)	17 (22.4%)	18 (30.0%)	
**Age (year)**	54.169 ± 11.765	55.066 ± 10.765	53.033 ± 12.926	0.319
**Peritumoral edema**				
Negative	91 (66.9%)	66 (86.8%)	25 (41.7%)	0.000
Positive	45 (33.1%)	10 (13.2%)	35 (58.3%)	
**Tumor size**				
<2cm	18 (13.2%)	16 (21.1%)	2 (3.3%)	0.000
2-5cm	95 (69.9%)	56 (73.7%)	39 (65.0%)	
>5cm	23 (16.9%)	4 (5.2%)	19 (32.7%)	
**Location**				
Parasinoidal	37 (27.2%)	16 (21.1%)	21 (35.0%)	0.001
Facies convexa	28 (20.6%)	16 (21.1%)	12 (20.0%)	
Skull base	24 (17.6%)	8 (10.5%)	16 (26.7%)	
Others	47 (34.6%)	36 (47.3%)	11 (18.3%)	
**Hypertension**				
Negative	113 (83.1%)	67 (88.2%)	46 (76.7%)	0.076
Positive	23 (16.9%)	9 (11.8%)	14 (23.3%)	
**Diabetes**				
Negative	132(97.1%)	74 (97.4%)	58 (96.7%)	0.810
Positive	4 (2.9%)	2 (2.6%)	2 (3.3%)	
**Epilepsy**				
Negative	134 (98.5%)	76 (100%)	58 (96.7%)	0.109
Positive	2 (1.5%)	0 (0%)	2 (3.3%)	

CEE, Cerebral edema exacerbation.

Categorical variables were presented as the number (percentage). Continuous variables consistent with a normal distribution were presented as mean ± standard deviation, otherwise the median and quartile are used. Chi-Square or Fisher Exact tests, as appropriate, were used to compare the differences in categorical variables, while the independent sample t-test was used to compare the differences in continuous variables.

As shown in **Table 3**, univariate analysis was used to determine the independent clinical risk variables for postoperative CEE in the primary cohort and the validation cohort, respectively. Similar to the previous results, in the primary cohort, we found a significant association between postoperative CEE and peritumoral edema (P = 0.000), tumor size (P = 0.000), and location (P = 0.018). In the validation cohort, peritumoral edema (P = 0.021) and location (P = 0.038) tended to be associated with postoperative CEE.

**Table 3:** Correlation between cerebral edema exacerbation and clinical characteristics of patients with meningioma in the primary cohort and validation cohort.

Characteristics	Primary cohort (n=90)		P-value	Validation cohort(n=46)		P-value
	Non-CEE	CEE		Non-CEE	CEE	
**No.**	55	35		21	25	
**Gender**						
Male	44 (80.0%)	26 (74.3%)	0.525	15 (71.4%)	16 (64.0%)	0.592
Female	11 (20.0%)	9 (25.7%)	6 (28.6%)	9 (36.0%)
**Age (year)**	53.473 ± 10.706	53.743 ± 12.183	0.912	59.240 ± 9.990	52.040 ± 14.096	0.056
**Peritumoral edema**						
Negative	49 (89.1%)	13 (37.1%)	0.000	17(81.0%)	12 (48.0%)	0.021
Positive	6 (10.9%)	22 (62.9%)	4 (19.0%)	13(52.0%)
**Tumor size**						
<2cm	11 (20.0%)	0 (0%)	0.000	5 (23.8%)	2 (8.0%)	0.100
2-5cm	42 (76.4%)	24 (68.6%)	14 (66.7%)	15 (60.0%)
>5cm	2 (3.6%)	11 (31.4%)	2 (9.5%)	8 (32.0%)
**Location**						
Parasinoidal	11 (20.0%)	12 (34.3%)	0.018	5 (23.8%)	9 (36.0%)	0.038
Facies convexa	11 (20.0%)	8 (22.8%)	5 (23.8%)	4 (16.0%)
Skull base	8 (14.5%)	10 (28.6%)	0 (0%)	6 (24.0%)
Others	25 (45.5%)	5 (14.3%)	11 (52.4%)	6 (24.0%)
**Hypertension**						
Negative	48 (87.3%)	26 (74.3%)	0.116	19 (90.5%)	20 (80.0%)	0.324
Positive	7 (12.7%)	9 (25.7%)	2 (9.5%)	5 (20.0%)
**Diabetes**						
Negative	53 (96.4%)	33 (94.3%)	0.641	21 (100%)	25 (100%)	a*
Positive	2 (3.6%)	2 (5.7%)	0 (0%)	0 (0%)
**Epilepsy**						
Negative	55 (100%)	34 (97.1%)	0.207	21 (100%)	24(96.0%)	0.354
Positive	0 (0%)	1 (2.9%)	0 (0%)	1 (4.0%)

CEE, Cerebral edema exacerbation. a* means no comparative significance.

Categorical variables were presented as the number (percentage). Continuous variables consistent with a normal distribution were presented as mean ± standard deviation, otherwise the median and quartile are used. Chi-Square or Fisher Exact tests, as appropriate, were used to compare the differences in categorical variables, while the independent sample t-test was used to compare the differences in continuous variables.

### Radiomics Feature Selection and Radiomics Signature Construction

**Figure 2:**
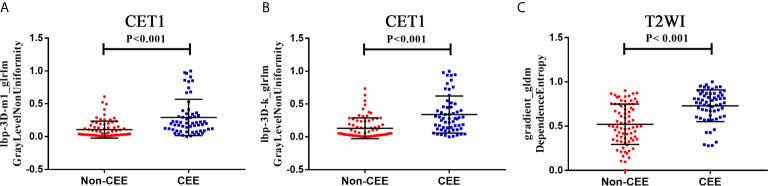
The selected three radiomics features showed significant differences between the postoperative CEE and non-CEE groups. **(A)** Texture feature ‘lbp-3D-m1_glrlm_GrayLevelNonUniformity’ in contrast-enhanced T1-weighted imaging sequence; **(B)** Texture feature ‘lbp-3D-k_glrlm_GrayLevelNonUniformity’ in contrast-enhanced T1-weighted imaging sequence; **(C)** Texture feature ‘gradient_gldm_DependenceEntropy’ in T2-weighted imaging sequence.

We extracted 3,124 radiomics features from one patient in two sequences. First, 1962 radiomics features were selected by Wilcoxon rank-sum test. Then, we use elastic net algorithm to determine 45 informative features. Finally, through the screening by RFE algorithm with 5-fold cross validation, 3 features that gave the best performance were selected as the final features for subsequent use. Two features were selected from the CET1 images, and one features from the T2WI images. The three selected radiomics features had significant differences in postoperative CEE and non-CEE groups (**Figure 2**, **Table 4**).

**Table 4:** The detail information of three selected radiomic features.

Sequence	Feature name	Feature type	Non-CEE	CEE	P value
CET1	lbp-3D-m1_glrlm_GrayLevelNonUniformity	Texture	0.03975 (0.0235-0.151)	0.2025 0.0938-0.3483)	<0.001
CET1	lbp-3D-k_glrlm_GrayLevelNonUniformity	Texture	0.05305 (0.0212-0.2258)	0.2820 (0.1215-0.4578)	<0.001
T2WI	gradient_gldm_DependenceEntropy	Texture	0.4970 (0.3500-0.7443)	0.7640 (0.6150-0.8648)	<0.001

CEE, Cerebral edema exacerbation; T2WI, T2-weighted imaging; CE-T1, contrast-enhanced T1-weighted imaging.

**Figure 3:**
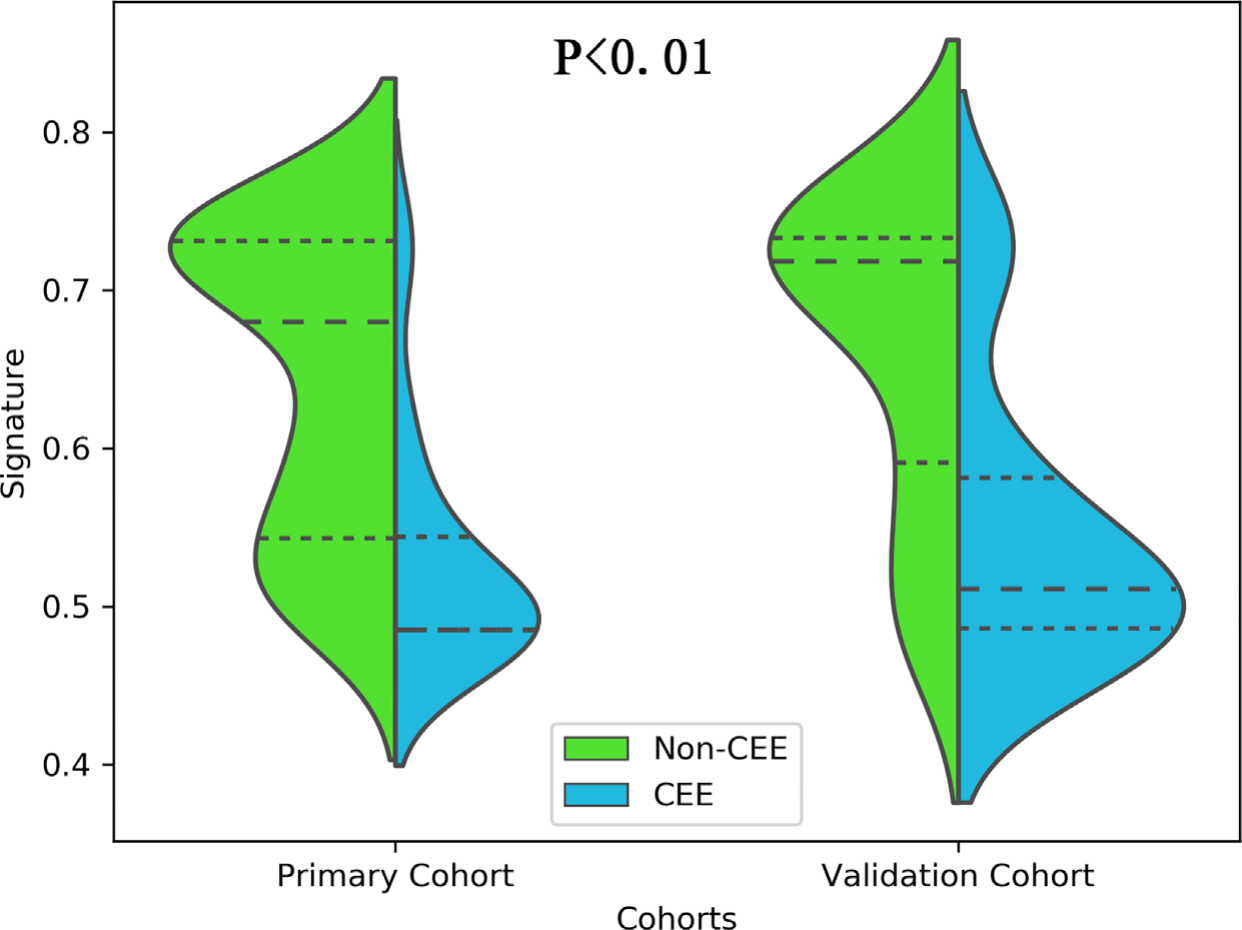
The distribution of the radiomics signature between postoperative CEE and non-CEE groups was compared by violin plot in the primary and validation cohorts.

**Figure 4:**
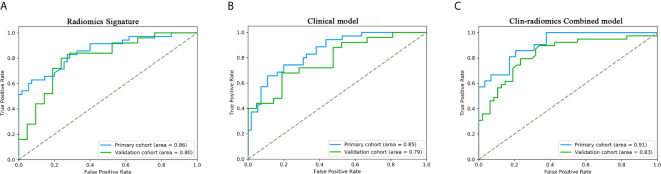
ROC curves of the radiomics signature, clinical model, and the clin-radiomics combined model in the primary and validation cohorts. The performances of these models were assessed using the AUC value. **(A)** Radiomics signature. **(B)** Clinical model. **(C)** Clin-radiomics combined model.

All 3 selected features were then entered into an SVM to build a radiomics signature. The violin plot showed significant differences in the distribution of the radiomics signature between postoperative CEE and non-CEE groups in both primary and validation cohorts (P<0.01; **Figure 3**). The radiomics signature showed favorable discrimination in predicting the postoperative CEE with AUC values of 0.86 (95% confidence interval [CI], 0.833–0.881) and 0.800(0.771-0.828) in the primary and validation cohorts, respectively (**Figure 4A**).

### Performance of Clinical and Clin-Radiomics Combined Model

The 8 available features in the primary cohort were used to build clinical model based on multivariable logistic regression analysis. We then verified the performance of these models in the validation cohort. As showed in **Figure 4B**, the AUCs were 0.85 (95% CI, 0.828-0.757) and 0.79 (95% CI, 0.757-0.815) in the primary and validation cohorts, respectively.

**Figure 5:**
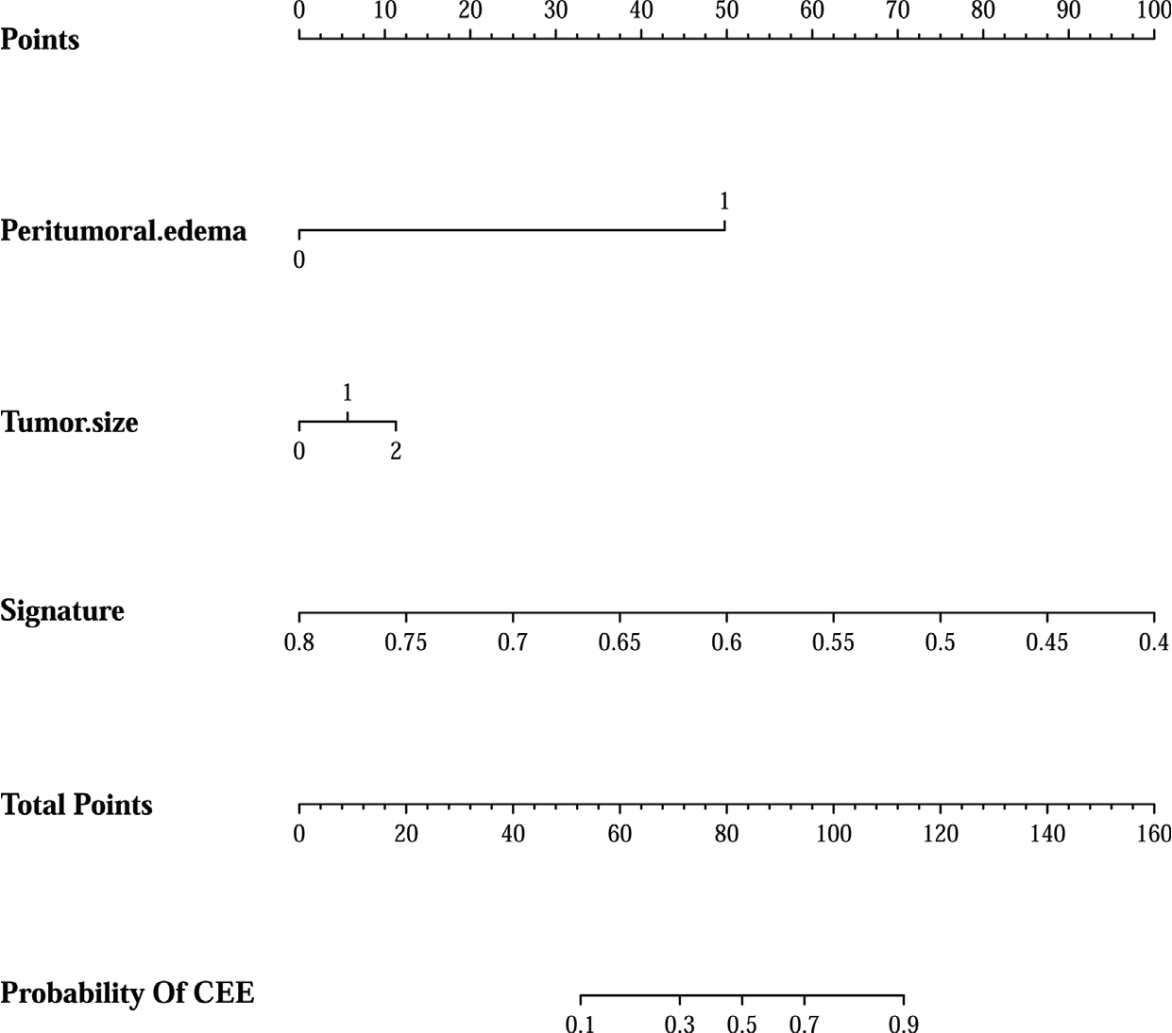
The clin-radiomics combined model is presented as a nomogram, which incorporated patients’ peritumoral edema, tumor size, and radiomics signature. The value of peritumoral edema, tumor size, and radiomics signature is located on corresponding lines 2-4, respectively. Draw a vertical line to the first line (point axis) to get the corresponding score. The total scores obtained for three included features are reflected in line 5 (total point axis), and the possibility of postoperative CEE has been determined in the last line.

In addition, after screening by AIC, two clinical characteristics (including peritumoral edema and tumor size) and radiomics signature were determined to establish the clin-radiomics combined model, yielded an AUC of 0.91 (95% CI, 0.893-926) in the primary cohort and 0.83 (95% CI, 0.808-0.858) in the validation cohort (**Figure 4C**). The predictive accuracy of the clin-radiomics combined model was 0.800 (0.775-0.824) in the primary cohort and 0.744 (0.718-0.770) in the validation cohort. The detailed predictive indicators of the three models are shown in **Table 5**. As showed in **Figure 5**, The clin-radiomics combined model is presented as a nomogram.

**Table 5:** Performance of radiomics signature, clinical model and combined model.

Model	Performance	AUC	ACC	SE	SP	PPV	NPV
Radiomic Signature	Primary cohort	0.86 (0.833-0.881)	0.7 (0.672-0.727)	0.857 (0.822-0.891)	0.6 (0.561-0.638)	0.577 (0.537-0.617)	0.868 (0.836-0.899)
	Validation cohort	0.800 (0.771-0.828)	0.761 (0.734-0.787)	0.84 (0.809-0.871)	0.667 (0.623-0.709)	0.75 (0.714-0.784)	0.778 (0.738-0.818)
Clinical model	Primary cohort	0.85 (0.828-0.757)	0.778 (0.751-0.804)	0.600 (0.552-0.650)	0.891 (0.866-0.915)	0.778 (0.732-0.823)	0.778 (0.747-0.810)
	Validation cohort	0.79 (0.757-0.815)	0.630 (0.600-0.660)	0.480 (0.438-0.520)	0.810 (0.772-0.845)	0.750 (0.703-0.780)	0.567 (0.529-0.604)
Combined model	Primary cohort	0.91 (0.893-926)	0.800 (0.775-0.824)	0.667 (0.621-0.710)	0.897 (0.872-0.921)	0.824 (0.782-0.864)	0.788 (0.757-0.818)
	Validation cohort	0.83 (0.808-0.858)	0.744 (0.718-0.770)	0.615 (0.572-0.659)	0.851 (0.822-0.880)	0.774 (0.733-0.816)	0.727 (0.693-0.761)

AUC, area under curve; ACC, accuracy; SN, sensitivity; SP, specificity; PPV, positive predict value; NPV, negative predictive value.

### Calibration and Clinical Usefulness Analysis

**Figure 6:**
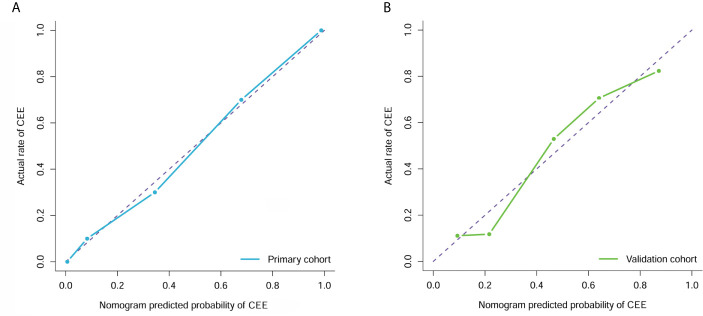
Calibration curve analysis for the clin-radiomics combined model in the primary **(A)** and validation **(B)** cohorts. Calibration curves depict the calibration of each model in terms of the agreement between the predicted and actual probability of the postoperative CEE probability. The Y axis represents the actual rate. The X axis represents the predicted probability. The diagonal purple line represents perfect prediction by an ideal model. The blue (primary cohort) and green (validation cohort) lines represent the performance of the clin-radiomics combined model, of which a closer fit to the diagonal purple line represents a better prediction.

The calibration curve analysis and Hosmer-Lemeshow test for clin-radiomics combined model demonstrated good agreement between observations and predictions in both the primary (P=0.95; **Figure 6A**) and validation cohorts (P=0.57; **Figure 6B**).

**Figure 7:**
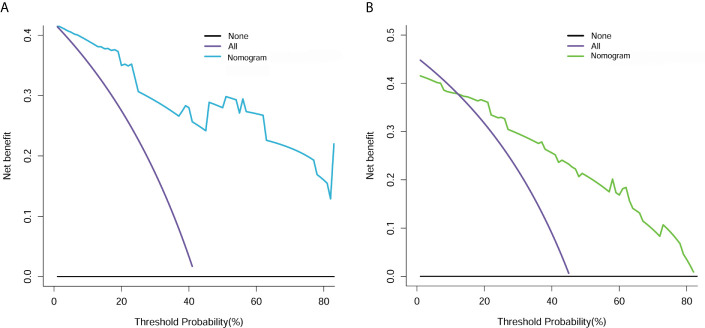
Decision curve analysis for clin-radiomics combined model in the primary **(A)** and validation **(B)** cohorts. The Y axis measures the net benefit. The blue (primary cohort) and green (validation cohort) lines represent the clin-radiomics combined model. The purple line represents the assumption that the postoperative CEE is highly expressed in all patients. The black line represents the assumption that no patients had a postoperative CEE.

The decision curve analysis for the clin-radiomics combined model is shown in **Figure 7**. The results showed that the clin-radiomics combined model performed a higher net benefit than both schemes, with a threshold probability of >0% for the primary cohort (**Figure 7A**) and a threshold probability of >13% for the validation cohort (**Figure 7B**). The results indicating that the clin-radiomics combined model were clinically useful.

## Discussion

Meningioma is a benign tumor originating from meningeal cells. It has the characteristics of high incidence rate, wide invasion area and high local recurrence rate. It seriously threatens people’s health and lives. It has attracted widespread attention in clinic (25). However, many patients have postoperative brain edema and severe life threatening. Therefore, it is particularly important to predict postoperative edema of meningioma. At present, most of the studies are about peritumoral edema of meningiomas before operation, and the mechanism is not completely clear. It may be related to the tumor itself factors, location, volume, pathological type (26), blood-brain barrier damage (27), endocrine activity and so on. However, there are few studies on the aggravation of postoperative brain edema. The edema of meningioma was mainly vascular origin, and the edema fluid was generated in tumor tissue. The formation mechanism of angiogenic brain edema is that the increase of capillary permeability leads to the infiltration of edema fluid and edema protein into peripheral brain tissue (28).Therefore, some scholars have shown that the aggravation of brain edema after surgery may be due to the further destruction of the structure adjacent to the blood-brain barrier, increased permeability, a large amount of water seeps from the capillary and accumulates in the extracellular space of nerve cells. It is undeniable that surgical injury is indeed one of the factors for aggravating brain edema (29).Some studies have also shown that brain edema after meningioma surgery is caused by the relief of tumor compression. When the compression is relieved, the blood-brain barrier is affected, which is easy to induce vasospasm and further aggravate the edema (30). Studies have shown that there is a significant correlation between the expression of vascular endothelial factor (31) and aquaporin-4 (32), which may be the factors of aggravating brain edema after surgery in patients with meningioma.

Radiomics is a new research field, which mainly through the extraction, processing and quantitative analysis of high-throughput data to explore the relationship with clinical value information (33). As described in our previous study and review (12), the radiomics process will first convert the radiographic images into the mineable data, which has involved 4 steps, namely, (a) image acquisition as well as reconstruction, (b) segmentation or labeling of the region of interest (ROI), (c) feature extraction as well as quantification, and (d) statistical analysis, establishment of the predictive and prognostic models. It has many applications in the central nervous system, such as differential diagnosis (34–37) and classification (15, 17), prediction of molecular characteristics (38, 39), therapeutic response and progress of central nervous system diseases (40, 41). These studies have shown that radiomics can be used to identify differences in treatment response, progression, and prognosis between patients with different CNS diseases, thus emphasizing that radiomics can be used as a new low-cost tool to improve treatment decisions for CNS diseases. Thus, we aim to develop an efficient and widely applicable preoperative radiomics model based on T2 and CET1 MRI images for predicting postoperative CEE in meningioma.

In the current study, Wilcoxon rank sum test, elastic net and RFE algorithm were sequentially utilized to reduce redundant features and select the most appropriate features for the construction of a radiomics signature. It is crucial to exclude irrelevant features, because these features may obscure important information and affect the performance of the prediction model (42). First, after the Wilcoxon rank sum test, we conducted a preliminary screening and got 1962 radiomics features. Then, 45 radiomics features were further obtained through the elastic net algorithm, and a feasible number that balances insufficient fitting and over fitting is obtained. Finally, using the RFE algorithm to select 3 features, a prediction model was constructed, and balanced performance was achieved in both the primary and validation cohorts.

Next, a radiomics signature and a clin-radiomics combined model that combined the radiomics signature and clinical features were constructed to predict the postoperative CEE of meningioma. The clin-radiomics combined model demonstrated a stable and reliable performance, reaching an AUC of 0.91 (95% CI, 0.893-926) and 0.83 (95% CI, 0.808-0.858), and an accuracy of 0.800 (0.775-0.824) and 0.744 (0.718-0.770) in the primary and validation cohorts, respectively. Good discrimination and good calibration were observed with the clin-radiomics combined model. The performance of the clin-radiomics combined model constructed is significantly higher than that of the clinical model, so the use of the clin-radiomics combined model is more accurate and more effective in predicting the postoperative brain edema and assisting clinical decision-making. These results indicated the reliability of the radiomics approach to non-invasively predict postoperative CEE in patients with meningioma.

This study has some limitations. First, this is a single-center retrospective study. Second, although internal patients were used to validate the model, the number of patients we included was small. Thus, more patients from multiple centers and prospective studies are necessary to verify the effectiveness and robustness of this clin-radiomics combined model. Finally, the standard of CEE adopted by us is as mentioned above, but different CEE standards will lead to different results.

## Conclusion

In conclusion, this retrospective study demonstrated that multiparametric MRI-based radiomics analysis is a promising approach for postoperative CEE prediction in patients with meningioma. It can serve as an effective noninvasive approach to predict postoperative CEE and determine individualized therapeutic schemes for patients with meningioma.

## Data availability statement

The raw data supporting the conclusions of this article will be made available by the authors, without undue reservation.

## Ethics Statement

The Ethical Review Committee of the Second Affiliated Hospital of Nanchang University approved the study design and protocol.

## Author Contributions

All authors contributed to the article and approved the submitted version. All authors analyzed and interpreted the data. BX and YF revised the manuscript for important intellectual content and contributed equally. ZW and XZ take final responsibility for this article.

## Funding

The present study was supported by Jiangxi provincial department of education natural science foundation youth project (grant no. GJJ180153), Science and technology plan of Jiangxi provincial health commission (grant no. 20201063).

## Conflict of Interest

The authors declare that the research was conducted in the absence of any commercial or financial relationships that could be construed as a potential conflict of interest.

## References

[1] Claus EB Bondy ML Schildkraut JM Wiemels JL Wrensch M Black PM. Epidemiology of intracranial meningioma. Neurosurgery (2005) 57(6):088–95. discussion -95. 10.1227/01.neu.0000188281.91351.b9 16331155

[2] Hasseleid BF Meling TR Ronning P Scheie D Helseth E. Surgery for convexity meningioma: Simpson Grade I resection as the goal: clinical article. J Neurosurg (2012) 117(6):999–1006. 10.3171/2012.9.JNS12294 23061394

[3] Gawlitza M Fiedler E Schob S Hoffmann KT Surov A. Peritumoral Brain Edema in Meningiomas Depends on Aquaporin-4 Expression and Not on Tumor Grade, Tumor Volume, Cell Count, or Ki-67 Labeling Index. Mol Imaging Biol (2017) 19(2):298–304. 10.1007/s11307-016-1000-7 27552812

[4] Berhouma M Jacquesson T Jouanneau E Cotton F. Pathogenesis of peri-tumoral edema in intracranial meningiomas. Neurosurg Rev (2019) 42(1):59–71. 10.1007/s10143-017-0897-x 28840371

[5] Galani V Lampri E Varouktsi A Alexiou G Mitselou A Kyritsis AP. Genetic and epigenetic alterations in meningiomas. Clin Neurol Neurosurg (2017) 158:119–25. 10.1016/j.clineuro.2017.05.002 28527972

[6] Palaniandy K Haspani MSM Zain NRM. Prediction of Histological Grade and Completeness of Resection of Intracranial Meningiomas: Role of Peritumoural Brain Edema. Malays J Med Sci (2017) 24(3):33–43. 10.21315/mjms2017.24.3.5 PMC5545616 28814931

[7] Asgari S Bassiouni H Hunold A Klassen D Stolke D Sandalcioglu IE. Extensive brain swelling with neurological deterioration after intracranial meningioma surgery - venous complication or ‘unspecific’ increase in tissue permeability. Zentralbl Neurochir (2008) 69(1):22–9. 10.1055/s-2007-992136 18393161

[8] Zhao Q Li S Tang Y Zhao C Xie M Li Z. Related factors of aggravated cerebral edema after meningioma surgery. J Regional Anat Operative Surg (2019) 28(01):55–9. 10.11659/jjssx.10E018066

[9] Lambin P Leijenaar RTH Deist TM Peerlings J de Jong EEC van Timmeren J. Radiomics: the bridge between medical imaging and personalized medicine. Nat Rev Clin Oncol (2017) 14(12):749–62. 10.1038/nrclinonc.2017.141 28975929

[10] Park JE Kim HS. Radiomics as a Quantitative Imaging Biomarker: Practical Considerations and the Current Standpoint in Neuro-oncologic Studies. Nucl Med Mol Imaging (2018) 52(2):99–108. 10.1007/s13139-017-0512-7 29662558 PMC5897262

[11] Chaddad A Kucharczyk MJ Daniel P Sabri S Jean-Claude BJ Niazi T. Radiomics in Glioblastoma: Current Status and Challenges Facing Clinical Implementation. Front Oncol (2019) 9:374. 10.3389/fonc.2019.00374 31165039 PMC6536622

[12] Fan Y Feng M Wang R. Application of Radiomics in Central Nervous System Diseases: a Systematic literature review. Clin Neurol Neurosurg (2019) 187:105565. 10.1016/j.clineuro.2019.105565 31670024

[13] Lu CF Hsu FT Hsieh KL Kao YJ Cheng SJ Hsu JB. Machine Learning-Based Radiomics for Molecular Subtyping of Gliomas. Clin Cancer Res (2018) 24(18):4429–36. 10.1158/1078-0432.CCR-17-3445 29789422

[14] van Griethuysen JJM Fedorov A Parmar C Hosny A Aucoin N Narayan V. Computational Radiomics System to Decode the Radiographic Phenotype. Cancer Res (2017) 77(21):e104–e7. 10.1158/0008-5472.CAN-17-0339 PMC5672828 29092951

[15] Fan Y Hua M Mou A Wu M Liu X Bao X. Preoperative Noninvasive Radiomics Approach Predicts Tumor Consistency in Patients With Acromegaly: Development and Multicenter Prospective Validation. Front Endocrinol (Lausanne) (2019) 10:403. 10.3389/fendo.2019.00403 31316464 PMC6611436

[16] Aerts HJ Velazquez ER Leijenaar RT Parmar C Grossmann P Carvalho S. Decoding tumour phenotype by noninvasive imaging using a quantitative radiomics approach. Nat Commun (2014) 5:4006. 10.1038/ncomms5644 24892406 PMC4059926

[17] Fan Y Chai Y Li K Fang H Mou A Feng S. Non-invasive and real-time proliferative activity estimation based on a quantitative radiomics approach for patients with acromegaly: a multicenter study. J Endocrinol Invest (2020) 43(6):755–65. 10.1007/s40618-019-01159-7 31849000

[18] Wang Q Li Q Mi R Ye H Zhang H Chen B. Radiomics Nomogram Building From Multiparametric MRI to Predict Grade in Patients With Glioma: A Cohort Study. J Magn Reson Imaging (2019) 49(3):825–33. 10.1002/jmri.26265 30260592

[19] Qu J Shen C Qin J Wang Z Liu Z Guo J. The MR radiomic signature can predict preoperative lymph node metastasis in patients with esophageal cancer. Eur Radiol (2019) 29(2):906–14. 10.1007/s00330-018-5583-z 30039220

[20] Zinn PO Singh SK Kotrotsou A Hassan I Thomas G Luedi MM. A Coclinical Radiogenomic Validation Study: Conserved Magnetic Resonance Radiomic Appearance of Periostin-Expressing Glioblastoma in Patients and Xenograft Models. Clin Cancer Res (2018) 24(24):6288–99. 10.1158/1078-0432.CCR-17-3420 PMC6538261 30054278

[21] Erturk SM. Receiver operating characteristic analysis. AJR Am J Roentgenol (2011) 197(4):W784. author reply W5. 10.2214/AJR.11.6484 21940556

[22] Pan W. Akaike’s information criterion in generalized estimating equations. Biometrics (2001) 57(1):120–5. 10.1111/j.0006-341X.2001.00120.x 11252586

[23] Kramer AA Zimmerman JE. Assessing the calibration of mortality benchmarks in critical care: The Hosmer-Lemeshow test revisited. Crit Care Med (2007) 35(9):2052–6. 10.1097/01.CCM.0000275267.64078.B0 17568333

[24] Vickers AJ Cronin AM Elkin EB Gonen M. Extensions to decision curve analysis, a novel method for evaluating diagnostic tests, prediction models and molecular markers. BMC Med Inform Decis Mak (2008) 8:53. 10.1186/1472-6947-8-53 19036144 PMC2611975

[25] Stepien K Ostrowski RP Matyja E. Hyperbaric oxygen as an adjunctive therapy in treatment of malignancies, including brain tumours. Med Oncol (2016) 33(9):101. 10.1007/s12032-016-0814-0 27485098 PMC4971045

[26] Go KG Wilmink JT Molenaar WM. Peritumoral brain edema associated with meningiomas. Neurosurgery (1988) 23(2):175–9. 10.1227/00006123-198808000-00008 3185876

[27] Bitzer M Topka H Morgalla M Friese S Wockel L Voigt K. Tumor-related venous obstruction and development of peritumoral brain edema in meningiomas. Neurosurgery (1998) 42(4):730–7. 10.1097/00006123-199804000-00029 9574636

[28] Klatzo I. Presidental address. Neuropathological aspects of brain edema. J Neuropathol Exp Neurol (1967) 26(1):1–14. 10.1097/00005072-196701000-00001 5336776

[29] Treggiari MM Schutz N Yanez ND Romand JA. Role of intracranial pressure values and patterns in predicting outcome in traumatic brain injury: a systematic review. Neurocrit Care (2007) 6(2):104–12. 10.1007/s12028-007-0012-1 17522793

[30] Tao C Wang J Zhang Y Qi S Liu F You C. Predictors of Acute Vertebrobasilar Vasospasm following Tumor Resection in the Foramen Magnum Region. PloS One (2016) 11(9):e0163908. 10.1371/journal.pone.0163908 27682826 PMC5040390

[31] Clement T Rodriguez-Grande B Badaut J. Aquaporins in brain edema. J Neurosci Res (2020) 98(1):9–18. 10.1002/jnr.24354 30430614

[32] Schob S Surov A Wienke A Meyer HJ Spielmann RP Fiedler E. Correlation Between Aquaporin 4 Expression and Different DWI Parameters in Grade I Meningioma. Mol Imaging Biol (2017) 19(1):138–42. 10.1007/s11307-016-0978-1 27357591

[33] Kermany DS Goldbaum M Cai W Valentim CCS Liang H Baxter SL. Identifying Medical Diagnoses and Treatable Diseases by Image-Based Deep Learning. Cell (2018) 172(5):1122–31 e9. 10.1016/j.cell.2018.02.010 29474911

[34] Kang D Park JE Kim YH Kim JH Oh JY Kim J. Diffusion radiomics as a diagnostic model for atypical manifestation of primary central nervous system lymphoma: development and multicenter external validation. Neuro Oncol (2018) 20(9):1251–61. 10.1093/neuonc/noy021 PMC6071659 29438500

[35] Artzi M Bressler I Ben Bashat D. Differentiation between glioblastoma, brain metastasis and subtypes using radiomics analysis. J Magn Reson Imaging (2019) 50(2):519–28. 10.1002/jmri.26643 30635952

[36] Qian Z Li Y Wang Y Li L Li R Wang K. Differentiation of glioblastoma from solitary brain metastases using radiomic machine-learning classifiers. Cancer Lett (2019) 451:128–35. 10.1016/j.canlet.2019.02.054 30878526

[37] Kim JY Park JE Jo Y Shim WH Nam SJ Kim JH. Incorporating diffusion- and perfusion-weighted MRI into a radiomics model improves diagnostic performance for pseudoprogression in glioblastoma patients. Neuro Oncol (2019) 21(3):404–14. 10.1093/neuonc/noy133 PMC6380413 30107606

[38] Zhou H Vallieres M Bai HX Su C Tang H Oldridge D. MRI features predict survival and molecular markers in diffuse lower-grade gliomas. Neuro Oncol (2017) 19(6):862–70. 10.1093/neuonc/now256 PMC5464433 28339588

[39] Xi YB Guo F Xu ZL Li C Wei W Tian P. Radiomics signature: A potential biomarker for the prediction of MGMT promoter methylation in glioblastoma. J Magn Reson Imaging (2018) 47(5):1380–7. 10.1002/jmri.25860 28926163

[40] Zhou M Scott J Chaudhury B Hall L Goldgof D Yeom KW. Radiomics in Brain Tumor: Image Assessment, Quantitative Feature Descriptors, and Machine-Learning Approaches. AJNR Am J Neuroradiol (2018) 39(2):208–16. 10.3174/ajnr.A5391 PMC5812810 28982791

[41] Fan Y Jiang S Hua M Feng S Feng M Wang R. Machine Learning-Based Radiomics Predicts Radiotherapeutic Response in Patients With Acromegaly. Front Endocrinol (Lausanne) (2019) 10:588. 10.3389/fendo.2019.00588 31507537 PMC6718446

[42] Niu J Zhang S Ma S Diao J Zhou W Tian J. Preoperative prediction of cavernous sinus invasion by pituitary adenomas using a radiomics method based on magnetic resonance images. Eur Radiol (2019) 29(3):1625–34. 10.1007/s00330-018-5725-3 PMC6510860 30255254

